# Factors Associated with Adequate Breastfeeding: Evidence from the Peruvian Demographic and Health Survey, 2019

**DOI:** 10.3390/ijerph192013607

**Published:** 2022-10-20

**Authors:** Mariela Yamunaque-Carranza, Sebastian A. Medina-Ramirez, Carlos S. Mamani-García, Brenda Caira-Chuquineyra, Daniel Fernandez-Guzman, Diego Urrunaga-Pastor, Guido Bendezu-Quispe

**Affiliations:** 1 Facultad de Medicina Humana, Universidad de San Martín de Porres, Chiclayo 14012, Peru; 2 Sociedad Científica de Estudiantes de Medicina Veritas (SCIEMVE), Chiclayo 14012, Peru; 3 Departamento de Ciencias Básicas, Facultad de Medicina Humana, Universidad Peruana Unión (UPeU), Lima 15464, Peru; 4 Facultad de Medicina, Universidad Nacional de San Agustín de Arequipa, Arequipa 04001, Peru; 5 Sociedad Científica de Estudiantes de Medicina Agustinos (SOCIEMA), Arequipa 04001, Peru; 6 Grupo Peruano de Investigación Epidemiológica, Unidad para la Generación y Síntesis de Evidencias en Salud, Universidad San Ignacio de Loyola, Lima 15074, Peru; 7 Escuela Profesional de Medicina Humana, Universidad Nacional de San Antonio Abad del Cusco, Cusco 08000, Peru; 8 Facultad de Ciencias de la Salud, Universidad Científica del Sur, Carrera de Medicina Humana, Lima 15067, Peru; 9 Centro de Investigación Epidemiológica en Salud Global, Universidad Privada Norbert Wiener, Lima 15046, Peru

**Keywords:** breastfeeding initiation, breastfeeding continuation, newborn, health surveys, Peru

## Abstract

Objective: To assess the factors associated with adequate breastfeeding (ABF) among Peruvian mothers during 2019. Materials and Methods: We performed a secondary analysis of the 2019 Demographic and Family Health Survey (ENDES) database of Peru. ABF was defined based on the recommendations of the World Health Organization, which defined it as starting breastfeeding within the first hour of life and continuing with exclusive breastfeeding for up to 6 months. To determine the factors associated with ABF, a Poisson generalized linear models with log-link function was used. Adjusted prevalence ratios (aPR) with their respective 95% confidence intervals (95% CI) were calculated. Results: A prevalence of ABF of 48.1% was identified among 11,157 women who reported at least one child in the last five years. Most of them were young (68.6%) and lived in urban areas (65.5%). Furthermore, being unemployed (aPR:1.02; 95% CI:1.00–1.04); residing on the coast, except for Metropolitan Lima (aPR:1.08; 95% CI:1.04–1.11), the highlands (aPR:1.14; 95% CI:1.11–1.18), and the jungle (aPR:1.20; 95% CI: 1.16–1.24); having had a vaginal delivery (aPR:1.30; 95% CI:1.27–1.05); and having two children (aPR:1.03; 95% CI:1.01–1.05) or three or more children (aPR:1.03; 95% CI:1.01–1.05) were associated with a higher frequency of ABF. Conclusions: One out of two women between 18–59 with children performed ABF. The factors associated with ABF were the current occupation, region of residence, type of delivery, and parity. Health policies and strategies should be implemented, considering our results, to promote maternal counseling by health personnel in order to increase the prevalence of ABF in the Peruvian population.

## 1. Introduction

Breastfeeding (BF) is recognized as a low-cost and effective intervention that guarantees newborns’ nutritional and health status [1]. Between 11.6% and 22.0% of infant mortality could be prevented with adequate breastfeeding (ABF) [2,3]. Late administration of BF and early introduction of food are associated with lower cognitive development in infants; an increased risk of sudden death; infant malnutrition; ear, gastrointestinal, and respiratory infections; atopies; and diabetes mellitus in the short and long term [4,5,6]. Moreover, ABF reduces the risk of hypertension, diabetes mellitus, obesity, breast cancer, and ovarian cancer in the mother [7].

The World Health Organization (WHO) and the American Academy of Pediatrics consider the initiation of BF within the first hour of life and maintaining it exclusively until the age of six months as indicators of exclusive breastfeeding (EBF) [2,8]. However, the global prevalence in 2018 of early initiation of breastfeeding (EIBF) within the first hour and EBF up to 6 months was 48% and 44%, respectively [9]. Therefore, both indicators were below the objective of global sustainable development, a goal that is expected to reach at least 70% by 2030 [10].

In high-income countries, such as the United States, approximately 81% of newborns received breastmilk at birth and 56% discontinue exclusive breastfeeding at three months [11]. In contrast, in low- and middle-income countries, the breastfeeding initiation rate in the first hour is lower [12]. In these countries, the initiation time between 0.5 and 40 hours after birth has been estimated [13]. In Latin America and the Caribbean, the prevalence of EIBF was 45.2–62.8% [14], while the prevalence of EBF up to 6 months was 63.0% [15].

Reportedly, in Peru, 49.7% of newborns started BF during the first hour of life, and 66.4% received EBF, according to the national health survey conducted in 2018 [16]. BF is a low-cost intervention with great benefits for the newborn and the mother; there is a need to identify the factors associated with ABF. This will be essential in formulating and promoting effective interventions, ensuring that mothers provide ABF, among the most vulnerable groups. Therefore, this study aimed to evaluate the factors associated with ABF among Peruvian mothers during 2019.

## 2. Materials and Methods

### 2.1. Study Design

We conducted a secondary analysis of the data, collected in the Demographic and Family Health Survey (ENDES, as per its Spanish acronym) of Peru in 2019. The ENDES is compiled annually through the National Institute of Statistics and Informatics (INEI by its Spanish acronym), and it includes three questionnaires—the “Household Questionnaire,” the “Questionnaire for Individual Women,”, and the “Health Questionnaire.” For this study, we only used the questionnaire for individual women, whose target population included women aged 15–49 years.

### 2.2. Population, Sample, and Sampling

ENDES is a multistage survey with probabilistic sampling by clusters and stratified at the departmental and urban–rural levels; thus, it is representative at urban–rural, regional, and national levels [17]. The primary sampling unit was the selected clusters, and the secondary sampling unit was the selected dwellings. Additional information on the ENDES survey methodology can be found in a prior technical report [17].

**Figure 1:**
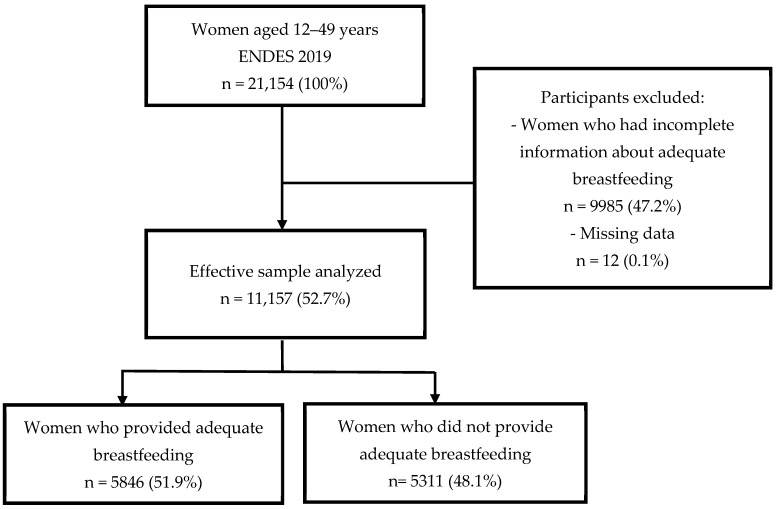
Flowchart of the selection of the study sample.

ENDES collected information from 33,289 women aged 12–49 years in 2019. In ENDES, only information regarding the child or children that a woman had had in the last five years is collected. This study used information on all the children who were older than six months old, i.e., the time required to assess whether BF was adequate. Respondents who did not meet all the variables of interest were excluded. After this screening, our effective sample was 11,157 mothers with their respective children (Figure 1).

### 2.3. Variables

The dependent variable was ABF collected through self-reporting. We defined this variable as adequate when BF started during the first hour after delivery and if it was maintained exclusively, without water intake or ingestion of other liquids or food for six months (180 days) [18].

The characteristics associated with BF described in the literature were considered covariates of the study [12,19]. We included characteristics of the mother, including age (adolescent [12–19 years], young [20–29 years], and adult [30–49 years]) according to the groups established by the INEI [20], region of origin (Metropolitan Lima, coastal regions, excluding Metropolitan Lima, highlands, and jungle), area of residence (urban and rural), educational level (primary or lower, secondary and higher), ethnic group (Quechua, mestizo or black, among others), occupation (employed or unemployed), wealth quintile (I–V), and affiliation to health insurance (yes or no). We also evaluated adequate prenatal care, which had to meet four conditions, as follows: first, having undergone at least six prenatal care sessions, according to the Ministry of Health of Peru [21]; second, having the first control during the first trimester; third, the fact that the care provided was quality care, and fourth, the fact that it had been performed by trained healthcare personnel. These conditions agree with those established by the WHO [22]. Furthermore, we included obstetric-gynecological characteristics, such as the number of children (first child, second child, and third child or more), type of pregnancy (single or multiple), place of delivery (institutional or non-institutional), and type of delivery (vaginal or cesarean section). We also considered whether the woman had received breastfeeding counseling (yes or no), defined as the educational process conducted by qualified health personnel in the area that could provide information on breastfeeding to pregnant women and postpartum women, which can take place in a health center or the community [23].

### 2.4. Statistical Analysis

We downloaded the ENDES 2019 databases and imported them into the Stata® v.17.0 statistical package (Stata Corporation, College Station, TX, USA). We performed the analyses considering the complex sampling of the survey by using the “svy” command.

The absolute frequencies and weighted proportions were calculated for the descriptive analysis of the categorical variables, and for the numerical variables, we estimated the means with the standard deviation. We evaluated the association between categorical variables for the bivariate analysis using the chi-square test with Rao–Scott correction.

To assess the associated factors, we used Poisson generalized linear models with log-link function, and crude prevalence ratios (cPR) and adjusted prevalence ratios (aPR) were calculated with their respective 95% confidence intervals (95% CI). For the adjusted model, the forward manual selection method and the Wald test were used to select the variables that allowed us to obtain a parsimonious final model. Therefore, the variables marital status, current occupation, region of residence, wealth index, parity, type of delivery, and place of delivery were introduced in the final model. We evaluated multicollinearity in the adjusted regression model using the variance inflation factor (VIF), where a value of >10 determined multicollinearity between variables. However, all the values obtained were <10. Furthermore, significance was indicated by *p* < 0.05 for all statistical tests.

## 3. Ethical Aspects

Approval by an ethics committee was not requested because this is an analysis of secondary data in the public domain. The ENDES data are coded without identifiers, so they do not allow the identification of the participants who were assessed. The database is freely accessible and can be downloaded from the INEI website (http://iinei.inei.gob.pe/microdatos/, accessed on 1 April 2022).

## 4. Results

Of the 21,154 women of reproductive age (12–49 years) who answered the women’s questionnaire in the ENDES 2019, 9985 were excluded for not meeting the exposure variable and 12 for lacking complete information in some of the covariates. Therefore, the final sample included 11,157 women (Figure 1).

### 4.1. General Characteristics of the Study Population

We determined that 68.6% (n = 7660) of the women were between 20 and 29 years of age, 85.7% (n = 9572) reported having a partner, 48.5% (n = 5504) reported having completed secondary education, 64.8% (n = 7393) were currently employed. Likewise, 31.9% (n = 4209) belonged to the highlands region of Peru, 65.5% (n = 6967) lived in urban areas, and 33.8% (n = 4174) belonged to the first wealth quintile. Likewise, 39.4% (n = 4524) of respondents reported having ≥3 children, 87.8% (n = 9974) gave birth at a health institution, and 83.1% (n = 9397) concluded in a vaginal delivery. After delivery, 67.8% (n = 7841) reported having breastfed their newborn within the first hour of life and 68.2% (n = 8067) reported that they provided EBF for at least 6 months. With this, 48.1% (n = 5846) of the women presented an ABF (Table 1).

### 4.2. Characteristics of the Study Population Based on Compliance with the ABF

The frequency of ABF was significantly more frequent among women who had a partner (49.2%; *p* < 0.001), those with a primary education level or less (57.7%; *p* < 0.001), women who were unemployed (49.9%; *p* = 0.049), women who lived in the jungle region of Peru (63.0%; *p* < 0.001) or in a rural area (59.5%; *p* < 0.001), women who belonged to the fifth wealth quintile (61.7%; *p* < 0.001), and women who identified themselves as belonging to Quechua ethnicity (52.2%; *p* < 0.001). For the variables related to pregnancy and childbirth, women who had ≥3 children (53.1%; *p* < 0.001), women with a singleton pregnancy (48.2%; *p* = 0.011), women who had a non-institutional delivery (58.3 %; *p* < 0.001), a vaginal delivery (54.7%; *p* < 0.001), and women who received BF counseling during pregnancy (49.7%; *p* < 0.001) presented a higher proportion of ABF (Table 2).

### 4.3. Factors Associated with ABF

In the multivariate regression analysis, we found that being unemployed (aPR: 1.02; 95% CI: 1.00–1.04), belonging to the coastal regions, with the exception of Lima (aPR: 1.08; 95% CI: 1.04–1.11), highlands (aPR: 1.14; 95% CI: 1.11–1.18), and jungle (aPR: 1.20; 95% CI: 1.16–1.24); and having had a vaginal delivery (aPR: 1.30; 95% CI: 1.27–1.32), and having two children (aPR: 1-03; 95% CI: 1.01–1.05) or three or more children (aPR: 1.03; 95% CI: 1.01–1.05) were associated with a higher frequency of presenting ABF (Table 3).

## 5. Discussion

### 5.1. Main Findings

This investigation evaluated the factors associated with ABF in a population of 11,157 women of reproductive age. We determined that approximately one out of two mothers offered ABF to their children. Being unemployed; living in the highlands, jungle, or coastal regions except for Metropolitan Lima; having had a vaginal delivery; and having two or more children were associated with a higher frequency of ABF. Health policies and maternal counseling by health personnel should be improved, considering the factors that were associated in our work, to promote in order to increase the prevalence of ABF in the Peruvian population.

### 5.2. The Frequency of ABF in Peru

The prevalence of ABF was 48.1% in Peru in 2019. Regarding the components of ABF, 67.8% practiced EIBF, a prevalence that exceeds the global prevalence for 2016 (45%) [24] and the prevalence reported in a systematic review conducted in 2013 that included studies from Brazil (11.4%) [12]. Similar to our finding, in Colombia a prevalence of IUF of 65.6% has been recorded; however, in their study they acknowledge that Colombia has been working for several years to create a breastfeeding-friendly environment [25]. In terms of EBF, we determined a frequency of 68.2%, which was higher than that reported worldwide (43%) in 2015 [23], and that reported in six Latin America and Caribbean countries, including Bolivia, Colombia, Dominican Republic, Guatemala, and Peru in 2010 (~50%) [26]. However, contrasting with more recent data from Latin American countries, our figures would be similar. In Chile there is a reported prevalence of EBF of 69.5% and in Ecuador a prevalence of 62.9% [27,28]. The prevalence of EBF in the population studied would be on track to reach the WHO’s global goal, which is to increase coverage to >70% by 2030 [10]. The higher prevalence of EIBF and EBF found compared to other countries of the region could be explained by the temporal difference with previous studies, which would influence a greater coverage of early breastfeeding immediately after delivery and the greater education of health personnel about the benefits of EBF and EIBF, that has been promoted worldwide and also because, in recent years, the Peruvian Ministry of Health is reinforcing and protocolizing technical guides of EBF [23]. 

### 5.3. Factors Associated with ABF

Regarding the factors associated with ABF, the regions of the highlands, jungle, and the rest of the coast, compared to Metropolitan Lima had a higher prevalence of ABF. Thus, the United Nations International Children’s Emergency Fund (UNICEF) warned of a decrease in EBF prevalence in Peruvian urban areas and areas with better income levels, such as Metropolitan Lima, in 2016 [29]. Furthermore, in Metropolitan Lima, the need to work was reportedly among the main reasons for early abandoning EBF [30]. This could be explained by the fact that there is a high frequency of informal jobs in this region [31]; thus, even pregnant women and new mothers would have to provide economic income. This situation could explain the findings of the investigation.

Regarding the current position (employed or unemployed), mothers without a job had a higher proportion of ABF. Likewise, the same association has been reported in other Latin American countries and in the United States [26,32,33]. This could be attributed to the fact that mothers without a job (formal or informal) would have the autonomy of their schedules to ensure exclusive breastfeeding for more months, while employed women needed to return to work early and would have less time to feed their children directly. It is reported that a higher proportion (by more than double) of unemployed mothers give EBF, compared to those who were employed [34,35]. The time offered (98 days, starting approximately 49 days before the expected date of delivery) could be insufficient, even though a maternity subsidy is provided in Peru [36]. Therefore, maternity leave could be insufficient for promoting or ensuring EBF for six months in the Peruvian context. Women with informal jobs would return to their long working hours, which in many cases are self-imposed to meet their family’s economic needs, a challenging situation for EBF. In this study, informality was not included in the analysis for ABF, since the ENDES database does not provide information regarding formal or informal job conditions. However, we consider it important to propose strategies to increase the awareness of ABF in mothers with informal jobs and policies to guarantee ABF in this population, since, as it is described in the literature, breastfeeding practices in women with informal jobs depend on the particular conditions of the women environment [37,38,39] On the other hand, maternity leave in those employed women should be reevaluated to guarantee ABF, taking into account the experiences of other countries where leave is extended for several months [40]. 

Our results indicate that the type of delivery is an important factor related to ABF, which is consistent with previous studies associated with EIBF [41,42]. It has also been reported that cesarean delivery is associated with a lower frequency of EIBF [12]. This could be because mothers who had a cesarean delivery presented late mother–child contact, which would also influence the physiology of lactogenesis and, therefore, the quality of breast milk [43]. Considering this, promoting ABF in all health centers with the capacity to attend births is important, especially among mothers giving birth through cesarean section. Considering its many advantages, vaginal delivery could be promoted for all cases at centers that primarily perform cesarean sections, thereby avoiding unjustified cases of cesarean sections. This is because cesarean sections occur in 3 out of every 10 deliveries in Peru [17], a value that is above the 10% recommended by the WHO [44]. 

Another factor associated with a higher frequency of presenting ABF was having two or more children. This finding was consistent with other reports in the literature [42]. This could be because, after the experience of delivering the first child, mothers were able to achieve a higher level of knowledge of BF. However, the biomedical literature has also reported the absence of a relationship between BF and parity [45], and one of the possible reasons is the influence of beliefs about BF in some regions and cultures [46]. Therefore, it is important to guarantee ABF, especially among first-time mothers, by promoting and strengthening the information provided during prenatal and postnatal care.

### 5.4. Relevance for Public Health and General Recommendation

BF has proven to be a low-cost practice, albeit with great benefits for the newborn’s and mother’s health [4,5,6,7]. The findings in this study indicate an important influence of sociodemographic and obstetric factors in relation to ABF among the Peruvian population.

Investment in BF, such as family-centered educational models, peer, and health care provider support to monitor breastfeeding, and implementation of technologies to improve communication in remote locations, continues to be limited in Peru, which could favor a higher rate of infant mortality, and financial losses in health [47]. It could also be why EIBF barely reached 50% in newborns at the national level by 2016. Likewise, BF would reach 70% among infants under six months, with a wide variation between regions—with the Tumbes region reporting the lowest (~30%) [48].

With the aim of meeting the goals of sustainable development—and given that ABF could mainly favor the improvement of nutrition, prevent infant mortality, reduce the risk of non-communicable diseases, and support cognitive development and education [4,5,6,7]—it is necessary to adequately promote and implement the recommendations made by international organizations, as well as by the Peruvian Ministry of Health, regarding ABF, considering the different scenarios and contexts in which this population operates.

Our findings indicate that approximately half of the mothers did not practice ABF, and sociodemographic characteristics influence this practice. Therefore, it is necessary to design or improve strategies according to these characteristics in order to promote ABF in the Peruvian population.

### 5.5. Limitations and Strengths

The following limitations in this study should be considered even though our results are consistent with those reported in previous studies [12,19,42]. First, we must recognize that, despite having found several related factors in our study, we cannot establish causality because there is no temporality due to the cross-sectional design of the survey. Second, since the data evaluated came from a secondary database, other factors related to childbirth or the child during the first months—such as conditions of the newborn (including prematurity, diseases that require hospitalization, morphological pathologies that conditions BF, such as muscle diseases, or those that contraindicated BF, such as galactosemia) or the mother (such as HIV infection)—that limited or contraindicated BF [49,50] were not included in the measurements made by the ENDES. Furthermore, we could not assess the third component of ABF (non-exclusive breastfeeding for up to 2 years), since this information is not available in the ENDES database. However, considering the recommendations and evaluations of BF, we consider that including only two components in the construct can similarly assess ABF. Third, there may have been recall bias or social desirability bias on the questionnaire questions collected through self-reporting. Finally, we consider that by not including in our outcomes other variables related to breastfeeding, such as mixed breastfeeding rate, full breastfeeding, and breastfeeding duration, could limit the understanding of this phenomenon and could be evaluated in future studies. Regardless of these limitations, the use of the database of this survey allows for the information to be evaluated with representativeness, since the ENDES is a national and regional survey, which is performed on an annual basis, and which has methodological quality control processes. Likewise, previous investigations in other countries have used demographic and health surveys, such as ENDES, to evaluate the sociodemographic factors related to BF. Therefore, this type of survey would be a useful source of data for the subject of interest. All things considered, we believe that the findings in this study can provide an overview of the factors associated with ABF among the Peruvian population.

## 6. Conclusions

Six out of ten infants received ABF. The main factors associated with a higher frequency of ABF were having a partner, being unemployed, belonging to regions outside Metropolitan Lima, as well as having had an institutional delivery, a vaginal delivery, and having two or more children. Therefore, it is necessary to implement strategies aimed at promoting and educating about ABF, considering our results (especially in the groups with less probability of presenting ABF). Additionally, to guarantee early breastfeeding, specific counseling could be integrated during prenatal visits to later promote exclusive breastfeeding during the puerperium and child growth and development controls, since these are low-cost and highly effective strategies to reduce morbidity in mother and child.

## Figures and Tables

**Table 1:** General characteristics of the study population (n = 11,157).

Characteristics	n	% *	95% CI *
**Age**			
Youth (20 to 29 years old)	7660	68.6	67.4–69.8
Teenagers (12 to 19 years old)	555	4.6	4.2–5.2
Adults (30 to 45 years old)	2942	26.8	25.6–28.0
**Marital status**			
Without partner	1585	14.3	13.5–15.2
With partner	9572	85.7	84.8–86.5
**Education level**			
Primary or lower	2965	26.2	25.0–27.5
Secondary	5504	48.5	47.1–49.8
Higher	2688	25.3	24.1–26.5
**Current occupation**			
Working	7393	64.8	63.3–66.2
Not working	3764	35.2	33.8–36.7
**Region**			
Metropolitan Lima	1122	24.2	23.0–25.5
Rest of the coast	2433	20.7	19.5–21.9
Highlands	4209	31.9	30.2–33.5
Jungle	3393	23.2	21.9–24.5
**Area of residence**			
Urban	6967	65.5	64.2–66.7
Rural	4190	34.5	33.3–35.8
**Wealth index**			
First quintile	4174	33.8	32.4–35.2
Second quintile	3260	26.9	25.6–28.2
Third quintile	1926	18.0	17.0–19.1
Fourth quintile	1164	13.1	12.2–14.1
Fifth quintile	633	8.2	7.4–9.0
**Ethnicity**			
Mestizo	4235	41.2	39.7–42.6
Quechua	3503	26.2	25.0–27.4
Black	1192	11.9	11.1–12.8
Others	2227	20.8	19.4–22.2
**Health insurance**			
Have	9522	83.2	82.1–84.2
Does not have	1635	16.8	15.8–17.9
**Type of gestation**			
Singleton	11,100	99.5	99.2–99.7
Multiple	57	0.5	0.3–0.8
**Type of delivery**			
Cesarean section	1760	16.9	15.9–17.9
Vaginal	9397	83.1	82.1–84.1
**Place of birth**			
Non-institutional	1183	12.2	11.1–13.5
Institutional	9974	87.8	86.5–88.9
**Parity**			
First child	3352	30.0	29.0–31.1
Second child	33,281	30.5	29.5–31.7
Third child or more	4524	39.4	38.2–40.7
**Adequate prenatal care**			
No	9609	86.1	85.2–87.0
Yes	1548	13.9	13.0–14.8
**Breastfeeding counseling during pregnancy**			
No	2795	27.2	25.9–28.5
Yes	8362	72.8	71.5–74.1
**Initiation of BF of the newborn**			
Greater than 1 hour	3316	32.2	30.9 - 33.5
Less than 1 hour	7841	67.8	66.5 - 69.1
**EBF up to before 6 months**			
Yes	8067	68.2	66.9–69.4
No	3090	31.8	30.6–33.1
**Adequate Breastfeeding**			
No	5311	51.9	50.6–53.2
Yes	5846	48.1	46.8–49.4

95% CI: Confidence interval at 95%. BF: breastfeeding. EBF: Exclusive breastfeeding. * Weighted values according to complex sampling of the survey.

**Table 2:** Prevalence of adequate breastfeeding, according to the characteristics of the study population (n = 11,157).

Characteristics	Adequate Breastfeeding						*p* Value **
	Yes			No			
	n	% *	95% CI *	n	% *	95% CI *	
**Age**							
Youth (20 to 29 years old)	4030	48.4	46.8*–*50.0	3630	51.6	50.0*–*53.2	0.529
Teenagers (12 to 19 years old)	299	49.7	44.3*–*55.1	256	50.3	44.9*–*55.7	
Adults (30 to 45 years old)	1517	47.0	44.5*–*49.5	1425	53.0	50.5*–*55.5	
**Marital status**							
Without partner	742	41.7	38.6*–*45.0	861	58.3	55.0*–*61.4	**<0.001**
With partner	5104	49.2	47.7*–*50.6	4450	50.8	49.4*–*52.3	
**Education level**							
Primary or lower	1785	57.7	55.2*–*60.1	1180	42.3	39.9*–*44.8	**<0.001**
Secondary	2923	48.7	46.9*–*50.4	2581	51.3	49.6*–*53.1	
Higher	1138	37.0	34.5*–*39.7	1550	63.0	60.3*–*65.5	
**Current occupation**							
Employed	3806	47.1	45.5*–*48.7	3587	52.9	51.3*–*54.5	0.049
Unemployed	2040	49.9	47.6*–*52.3	1724	50.1	47.7*–*52.4	
**Region**							
Metropolitan Lima	310	28.6	25.4*–*32.1	812	71.4	67.9*–*74.6	**<0.001**
Rest of the coast	1038	42.5	39.7*–*45.3	1395	57.5	54.7*–*60.3	
Highlands	2397	55.7	53.6*–*57.7	1812	44.3	42.3*–*46.4	
Jungle	2101	63.0	60.6*–*65.3	1292	37.0	34.7*–*39.4	
**Area of residence**							
Urban	3300	42.1	40.4*–*43.7	3667	57.9	56.3*–*59.6	**<0.001**
Rural	2546	59.5	57.3*–*61.6	1644	40.5	38.4*–*42.7	
**Wealth index**							
First quintile	225	32.3	27.7*–*37.2	408	67.7	62.8*–*72.3	**<0.001**
Second quintile	427	34.2	30.6*–*38.1	737	65.8	61.9*–*69.4	
Third quintile	863	40.2	37.2*–*43.3	1063	59.8	56.7*–*62.8	
Fourth quintile	1696	47.9	45.4*–*50.3	1564	52.1	49.7*–*54.6	
Fifth quintile	2635	61.7	59.5*–*63.7	1539	38.3	36.3*–*40.5	
**Ethnicity**							
Half Blood	2068	44.1	42.0*–*46.2	2167	55.9	53.8*–*58.0	**<0.001**
Quechua	1985	52.2	50.0*–*54.4	1518	47.8	45.6*–*50.0	
Black	633	51.0	47.4*–*54.7	559	49.0	45.3*–*52.6	
Others	1160	49.1	45.9*–*52.4	1067	50.9	47.6*–*54.1	
**Health insurance**							
Had	5060	48.5	47.1*–*49.9	4462	51.5	50.1*–*52.9	0.189
Did not had	786	46.0	42.5*–*49.5	849	54.0	50.5*–*57.5	
**Type of gestation**							
Singleton	5832	48.2	46.9*–*49.5	5268	51.8	50.5*–*53.1	**0.011**
Multiple	14	24.9	12.7*–*43.2	43	75.1	56.8*–*87.3	
**Type of delivery**							
Cesarean section	301	15.2	13.2*–*17.6	1459	84.8	82.4*–*86.8	**<0.001**
Vaginal	5545	54.7	53.3*–*56.2	3852	45.3	43.8*–*46.7	
**Place of birth**							
Non-institutional	708	58.3	54.0*–*62.5	475	41.7	37.5*–*46.0	**<0.001**
Institutional	5138	46.7	45.3*–*48.0	4836	53.3	52.0*–*54.7	
**Parity**							
First child	1583	42.9	40.7*–*45.1	1769	57.1	54.9*–*59.3	**<0.001**
Second child	1678	46.7	44.4*–*49.0	1603	53.3	51.0*–*55.6	
Third child or more	2585	53.1	51.2*–*55.1	1939	46.9	44.9*–*48.8	
**Adequate prenatal care**							
No	5047	48.1	46.7*–*49.6	4562	51.9	50.4*–*53.3	0.431
Yes	799	47.8	44.5*–*51.1	749	52.2	48.9*–*55.5	
**Breastfeeding counseling during pregnancy**							
No	1360	43.8	41.1*–*46.4	1435	56.2	53.6*–*58.9	**<0.001**
Yes	4486	49.7	48.2*–*51.2	3876	50.3	48.8*–*51.8	

* Percentages weighted according to the complex sampling of the survey. ** Calculated by chi^2^ independence test with Rao–Scott correction for complex sampling. *p*-values < 0.05 are in bold.

**Table 3:** Factors associated with adequate breastfeeding.

Characteristics	Adequate Breastfeeding					
	Crude Model			Parsimonious Fitted Model		
	cPR	95% CI	*p* Value	aPR	95% CI	*p* Value
**Current occupation**						
Employed	Ref.			Ref.		
Unemployed	1.02	1.00*–*1.04	0.048	1.02	1.00*–*1.04	**0.040**
**Region**						
Metropolitan Lima	Ref.			Ref.		
Rest of the coast	1.11	1.07*–*1.14	**<0.001**	1.08	1.04*–*1.11	**<0.001**
Highlands	1.21	1.18*–*1.25	**<0.001**	1.14	1.11*–*1.18	**<0.001**
Jungle	1.27	1.23*–*1.31	**<0.001**	1.20	1.16*–*1.24	**<0.001**
**Wealth index**						
First quintile	Ref.			Ref.		
Second quintile	1.01	0.97*–*1.06	0.528	0.98	0.94*–*1.03	0.511
Third quintile	1.06	1.02*–*1.11	**0.008**	0.98	0.94*–*1.03	0.455
Fourth quintile	1.12	1.07*–*1.16	**<0.001**	1.00	0.96*–*1.04	0.873
Fifth quintile	1.22	1.18*–*1.27	**<0.001**	1.03	0.99*–*1.07	0.166
**Type of delivery**						
Cesarean section	Ref.			Ref.		
Vaginal	1.34	1.32*–*1.37	**<0.001**	1.30	1.27*–*1.32	**<0.001**
**Parity**						
First child	Ref.			Ref.		
Second child	1.03	1.01*–*1.05	**0.015**	1.03	1.01*–*1.05	**0.002**
Third child or more	1.07	1.05*–*1.09	**<0.001**	1.03	1.01*–*1.05	**0.002**

PR: Prevalence Ratio. 95% CI: Confidence interval at 95%. Prevalence ratios and confidence intervals were calculated considering the complex sampling of the survey. *p*-values < 0.05 are in bold.

## Data Availability

The database used is in the public domain (http://iinei.inei.gob.pe/microdatos/, accessed on 1 April 2022). No registration is needed to download the ENDES database.
